# Dry Liposuction for Upper-Extremity End-Stage Lymphedema: A Step-by-Step Video of Technique

**DOI:** 10.1007/s00266-026-05745-y

**Published:** 2026-03-17

**Authors:** Martin Sollie, Caroline Lilja, Christoffer Bing Ydo, Thomas Foged, Jens Ahm Sørensen, Jørn Bo Thomsen

**Affiliations:** 1 Research Unit for Plastic Surgery, Odense University Hospital, Odense, Denmark; 2 Department of Plastic Surgery, Odense University Hospital, Odense, Denmark; 3 Present Address: Department of Plastic and Reconstructive Surgery, Oslo University Hospital, Sognsvannsveien 20, 0372 Oslo, Norway

**Keywords:** Lymphedema, Lipedema, Liposuction, Extremities

## Abstract

**Abstract:**

Lymphedema is a common, debilitating condition that challenges treatment efforts. The primary aim of treatment is to debulk the affected areas, providing relief without further damaging tissues or causing undesirable cosmetic outcomes. Liposuction has become a popular treatment for end-stage extremity lymphedema, yet there is no consensus on the most effective technique. Current practices include a range of methods such as dry, wet, superwet, laser-assisted, water-assisted, and power-assisted liposuction. Dry liposuction has sparked controversy, primarily due to concerns that it might be less gentle, and therefore potentially more harmful, than wet liposuction. Contrary to this, we posit that dry liposuction is a viable, low-risk alternative that minimizes the risk of tissue and lymph vessel damage while providing excellent and durable outcomes. This assertion is supported by current literature. In this video article, we present our systematic, step-by-step approach to performing dry liposuction for end-stage lymphedema, illustrating its efficacy and safety. Our personal experience is that patients experience significant reductions in limb volume and report high satisfaction rates, demonstrating that dry liposuction is both effective and reliable for the long-term management of end-stage lymphedema.

**Level of Evidence V:**

This journal requires that authors assign a level of evidence to each article. For a full description of these Evidence-Based Medicine ratings, please refer to the Table of Contents or the online Instructions to Authors  www.springer.com/00266

**Supplementary Information:**

The online version contains supplementary material available at 10.1007/s00266-026-05745-y.

## Introduction

Lymphedema is characterized by localized fluid retention and tissue swelling, which can arise from various causes. In the Western world, it is predominantly a consequence of surgical interventions for cancer, with breast cancer treatments being the most prevalent contributor, affecting 9% to 40% of patients [1, 2].

**Fig. 1:**
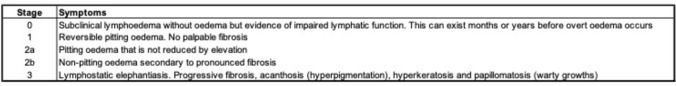
Staging of lymphedema according to the International Society of Lymphology

The severity of lymphedema spans a spectrum, from asymptomatic cases to severe manifestations with profound physical and psychological repercussions [3]. Clinical classification and staging of lymphedematous swelling have been defined by the International Society of Lymphology (Fig. 1) [4]. Initial management typically involves compression therapy, targeting symptom alleviation and disease progression prevention [5]. As the lymphedema progresses, the limb becomes fibrotic, resulting in non-pitting edema [6]. It is also hypothesized that lymphedema can stimulate adipose tissue growth, leading to disease progression even with optimal therapeutic interventions. Consequently, managing lymphedema remains challenging as the limb continues to increase in size. Therefore, in advanced cases, surgical debulking or liposuction may emerge as the sole viable treatment options [7].

Liposuction has proved to be a good option for these patients for several decades, providing relief, decreased risk of infections such as erysipelas, and good long-lasting results [8, 9]. Several studies have also reported significant improvements in patients' quality of life [10, 11]. The choice of optimal liposuction method is still debated, with tumescent liposuction arguably being the most commonly used. In our practice, we use dry liposuction for these patients, which we believe may be advantageous compared to other methods.

In this paper, we present our step-by-step method of dry liposuction for treating end-stage lymphedema. We have treated over fifty patients using this method. Our method is based on the methods published by Brorson et al. numerous times [12].

## The Technique

Dry liposuction is performed without the use of a tumescent solution. A bloodless surgical field is facilitated by a tourniquet around the proximal part of the extremity. The limb can then be systematically treated/liposuctioned to reach the desired results. The surgery is relatively fast as there is no need to allocate time for the application of a tumescent solution. Compared to wet techniques, dry liposuction has the great advantage of enabling accurate perioperative evaluation of the surgical outcome. There also seems to be less bleeding, and postoperative swelling is compared to tumescent techniques.

One of the drawbacks of the method is that it must be done under general anesthesia, due to the lack of tumescent anesthetic fluid and the use of a tourniquet. The full length of the extremity cannot be treated with the tourniquet as the most proximal part of the limb where the tourniquet is placed must be treated separately in the last stage of the surgery. For this purpose, this area of the extremity is treated with tumescent liposuction to finalize surgery. The tumescent liquid used is the formulation first described by Dr. Klein [13].

After liposuction compression garments are often used to ensure the best possible long-term results. It is debated among physicians whether compression treatments are a debilitating factor for the patients. Studies have, however, indicated that compression treatment following liposuction seems to improve the long-term results [14]. The patients stay admitted for two nights before being discharged to their own home.

## Preoperative Assessment

To be eligible for lymphedema surgery within our public health care system, the following requirements must be met:

1. **Exclusion of Cancer Recurrence**: Recurrence of any previous cancer must be clinically and radiographically excluded. For example, a clinical mammogram must be available in cases of previous breast cancer.
2. **Non-Pitting Lymphedema**: The patient's lymphedema must be of the 'non-pitting' type. Suggested examination: Press the thumb firmly into the skin in the middle of the forearm. Maintain the pressure for one minute. Measure the depth of the indentation. Repeat the examination on the middle of the upper arm. If the measured depth is <3-4 mm, treatment can be offered.
3. **Optimal Compression Therapy**: The patient must have already been treated with optimal compression therapy by a lymphedema therapist, and the therapist must have assessed that further treatment will not have additional effects.
4. **Commitment to Postoperative Care**: The patient must be prepared to use compression sleeves and possibly gloves around the clock for life after treatment.

## Surgical Technique—Step-by-Step

### Step 1: Preoperative Markings

**Fig. 2:**
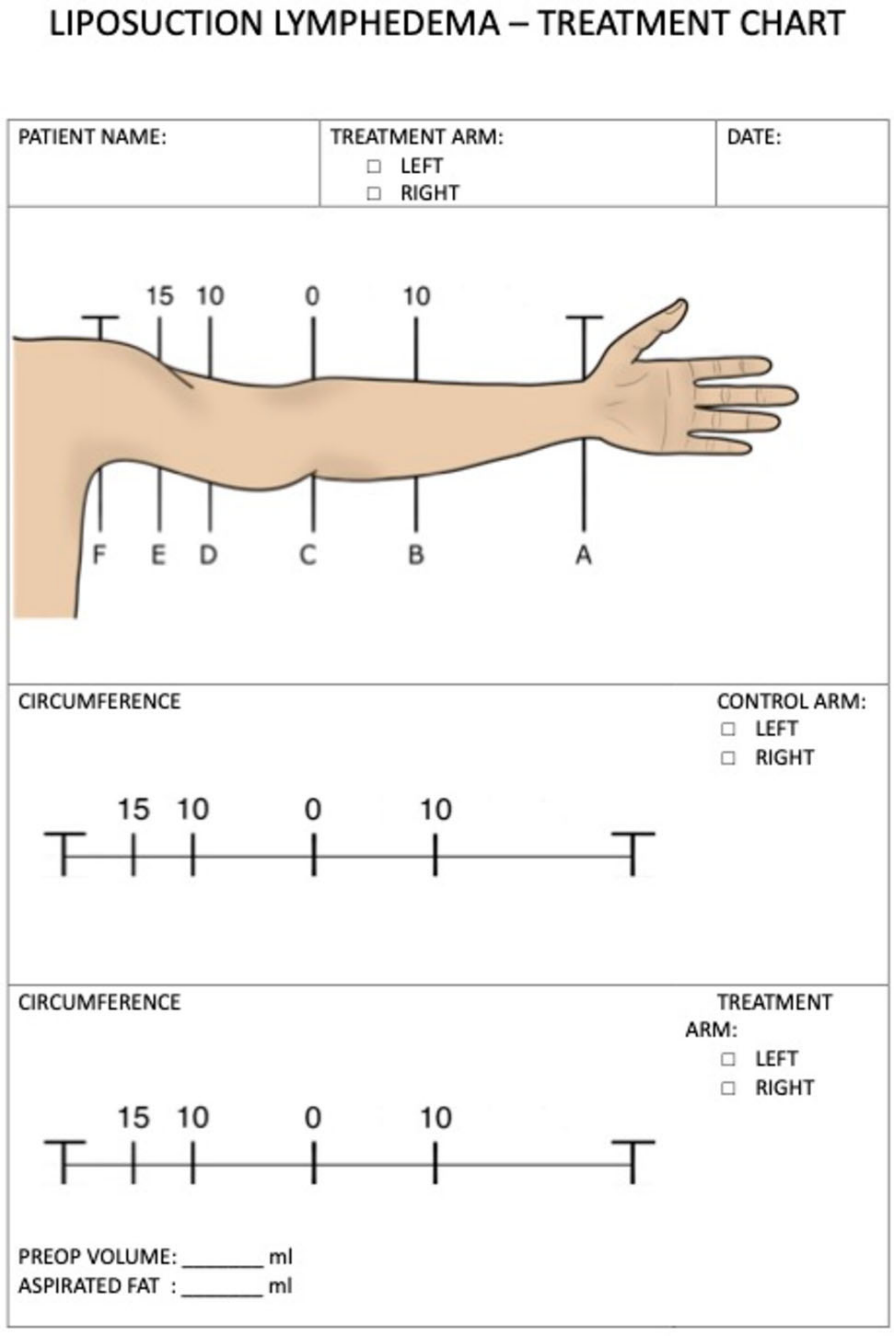
Lymphedema treatment chart: used for preoperative and intraoperative monitoring. Full chart in appendix

On the day of surgery, both the lymphedema arm and the healthy extremity are examined and measured, as the healthy will act as control during surgery. All measurements are registered on our premade form (Fig. 2).

### Step 2: Application of Tourniquet

A tourniquet is applied to allow for treatment in a bloodless field. Blood is forced out of the extremity using an Esmarch bandage. The tourniquet is then inflated to 250 mmHg.

### Step 3: Dry Liposuction—A Systematic Approach

The limb is treated with liposuction in a structured manner, beginning at the most distal part. Using multiple small incisions, the entire limb is treated. The diameters of the cannulas used are 3mm and 4mm. To monitor progress, the volume of the arm is measured and compared to the other, healthy limb. Liposuction is completed when the surgeon is satisfied with the results. We use the Eva Sp®6 machine (Euromi, Zoning industriel des Plenesses, Belgium) for liposuction with the following settings: Aspiration, 3.6 Bars (Control) & 0.9 Bar (Aspi).

### Step 4: Tumescent/Wet Liposuction

The most proximal part of the arm is not accessible for dry liposuction due to the tourniquet. This small area is therefore treated using conventional tumescent liposuction. The lipoaspirate is collected for both the dry and wet liposuction and is left to sediment overnight. The total volume aspirated fat is then recorded the day after the procedure. The volume typically ranges between 2 and 4 liters of fat per procedure.

### Step 5: Compression Treatment

After the treatment of liposuction, the patient is fitted with a compression garment to minimize bleeding and allow for excess fluid drainage. We recommend that all patients use lifelong compression garments rated as medical grade 4 (36+ mmHg). Some patients report that they, with time, can have shorter periods without compression (typically 2-3 hours) without it giving them any discomfort. The patients are admitted for two nights after the procedure before they are discharged.

## Discussion

End-stage upper-extremity lymphedema remains difficult to manage once conservative treatment has failed and the limb has progressed to a non-pitting, fibrotic state. In such cases, liposuction-based debulking has become an established surgical option, although the optimal technique remains debated.

In this video article, we present a systematic approach to dry liposuction performed under tourniquet control. When applied to carefully selected patients, this method allows for precise intraoperative assessment of volume reduction without the confounding effects of tumescent infiltration. In our experience, the technique is efficient, associated with acceptable pain levels, and results in limited postoperative swelling and a low complication rate, comparable to conventional tumescent liposuction.

Patient heterogeneity is a key consideration in lymphedema surgery. Disease etiology, duration, and degree of fibrosis influence both technical complexity and achievable outcomes. In cases with extensive fibrotic tissue, surgical expectations must be adjusted accordingly. Patient selection in our practice is primarily based on clinical assessment, particularly the presence of non-pitting edema. Some clinicians also use ultrasound as a method of selecting the most appropriate patients, but we have not seen this as a necessity in our clinic as even the more fibrotic tissues can be reduced.

We believe that postoperative compression therapy remains essential for maintaining long-term results following liposuction for lymphedema. Although lifelong compression may be perceived as burdensome, it plays a crucial role in preventing recurrence and preserving volume reduction.

The main limitation of this study is its descriptive nature and lack of standardized outcome measures or a comparative cohort. Nevertheless, the strength of this article lies in its practical, step-by-step video-based presentation of a reproducible surgical technique based on established principles and long-term clinical experience.

## Conclusion

We believe that dry liposuction is an easy, effective, and reliable method for treating end-stage lymphedema, as it reduces both limb circumference and volume together with patients reporting improvement in quality of life. We hope this visualized paper will inspire and guide other surgeons when treating these patients.

## Supplementary Information

Below is the link to the electronic supplementary material.

Supplementary file1 (MP4 378954 kb)
